# Gaze During Locomotion in Virtual Reality and the Real World

**DOI:** 10.3389/fnins.2021.656913

**Published:** 2021-05-24

**Authors:** Jan Drewes, Sascha Feder, Wolfgang Einhäuser

**Affiliations:** ^1^Institute of Brain and Psychological Sciences, Sichuan Normal University, Chengdu, China; ^2^Physics of Cognition Group, Institute of Physics, Chemnitz University of Technology, Chemnitz, Germany; ^3^Cognitive Systems Lab, Institute of Physics, Chemnitz University of Technology, Chemnitz, Germany

**Keywords:** gaze, eye tracking, virtual reality, real world, virtual locomotion

## Abstract

How vision guides gaze in realistic settings has been researched for decades. Human gaze behavior is typically measured in laboratory settings that are well controlled but feature-reduced and movement-constrained, in sharp contrast to real-life gaze control that combines eye, head, and body movements. Previous real-world research has shown environmental factors such as terrain difficulty to affect gaze; however, real-world settings are difficult to control or replicate. Virtual reality (VR) offers the experimental control of a laboratory, yet approximates freedom and visual complexity of the real world (RW). We measured gaze data in 8 healthy young adults during walking in the RW and simulated locomotion in VR. Participants walked along a pre-defined path inside an office building, which included different terrains such as long corridors and flights of stairs. In VR, participants followed the same path in a detailed virtual reconstruction of the building. We devised a novel hybrid control strategy for movement in VR: participants did not actually translate: forward movements were controlled by a hand-held device, rotational movements were executed physically and transferred to the VR. We found significant effects of terrain type (flat corridor, staircase up, and staircase down) on gaze direction, on the spatial spread of gaze direction, and on the angular distribution of gaze-direction changes. The factor world (RW and VR) affected the angular distribution of gaze-direction changes, saccade frequency, and head-centered vertical gaze direction. The latter effect vanished when referencing gaze to a world-fixed coordinate system, and was likely due to specifics of headset placement, which cannot confound any other analyzed measure. Importantly, we did not observe a significant interaction between the factors world and terrain for any of the tested measures. This indicates that differences between terrain types are not modulated by the world. The overall dwell time on navigational markers did not differ between worlds. The similar dependence of gaze behavior on terrain in the RW and in VR indicates that our VR captures real-world constraints remarkably well. High-fidelity VR combined with naturalistic movement control therefore has the potential to narrow the gap between the experimental control of a lab and ecologically valid settings.

## Introduction

The question what guides our gaze in realistic settings has been of interest to researchers for decades. Since the pioneering studies of Buswell (1935) and Yarbus (1967), this issue has long been reduced to eye movements during scene viewing; that is, observers looking at pictures of natural scenes with little to no head and body movements. Since the turn of the millennium, many computational models that predict gaze allocation for such scene viewing have been developed. Since, Itti et al. (1998) adapted Koch and Ullman (1985) “saliency map” to predict fixated locations in a natural scene, many models followed the idea to combine (low-level) image features using increasingly sophisticated schemes or optimality principles (e.g., Bruce and Tsotsos, 2006; Harel et al., 2006; Zhang et al., 2008; Garcia-Diaz et al., 2012). As such models presumably built-in some implicit (proto-)object representation and objects are crucial for gaze guidance (Stoll et al., 2015), it comes as no surprise that models that use deep neural networks that share their lower-levels with object recognition models (e.g., Kümmerer et al., 2015), have become most successful and close to the theoretical image-computable optimum in predicting gaze during free viewing of natural scenes. However, such image-computable models do not explicitly include other factors that are crucial for gaze guidance in natural scenes (Tatler et al., 2011), such as the task (Buswell, 1935; Yarbus, 1967; Hayhoe and Ballard, 2005; Underwood and Foulsham, 2006; Henderson et al., 2007; Einhäuser et al., 2008), semantics (Henderson and Hayes, 2017) or interindividual differences (de Haas et al., 2019). Crucially, most modeling and experimental studies alike, have used scene viewing with the head-fixed, which provides good experimental control, but the transfer to real-world scenarios is less clear.

In typical laboratory settings, where the movement of head and body is highly constrained, eye movements typically consist mainly of saccades – rapid shifts of gaze – and fixations – times in which the eyes are relatively stable and only small fixational eye movements [drift, microsaccades and tremor, Rolfs (2009) and Martinez-Conde et al. (2004) for reviews] occur. When a target moves though the visual field, it can be followed by smooth pursuit eye movements (Ilg, 2002; Spering and Montagnini, 2011); when the whole visual field moves, an optokinetic nystagmus is induced that stabilizes the image on the retina through slow eye-movement phases, whose dynamics is similar to pursuit (Magnusson et al., 1986), and resets the eyes in their orbit by fast phases, whose dynamics is similar to saccades (e.g., Garbutt et al., 2001). If the head is moved, the vestibular ocular reflex (VOR) quickly stabilizes gaze by counterrotating the eyes relative to the head (Fetter, 2007). While these classes of eye movements can be distinguished based on their dynamics and use in part different neuronal circuitry (Ilg, 1997; Kowler, 2011 for reviews), during real-world behaviors these movements interact and their conceptual separation becomes less clear (Steinman and Collewijn, 1980). For example, if an observer tracks an object that is stationary in the world while they are moving in the world, conceptually, this would be close to a fixation, while the eyes are clearly moving relative to their orbit. Hence for complex scenarios it is often critical to carefully distinguish between separate coordinate systems (e.g., eye movements relative to the head – hereafter referred to as “eye-in-head,” head movements relative to the world – “head in world,” or eye movements relative to the world, hereafter “gaze-in-world”) and to consider variables of interest, such as eye movement velocity in either coordinate frame, rather as a continuum than as means of distinguishing eye-movement classes strictly. Nonetheless, we still identify saccades based on velocity criteria (Engbert and Kliegl, 2003) for analysis purposes, while we do not separate any other classes further. Besides the mentioned convergent eye movements (both eyes move in unison), there are also divergent (vergence) eye movements, which we do not consider here, as in most cases objects of interest are at a considerable distance making the size of vergence movements small to negligible relative to other movements.

Even without an explicit task, participants exploring the real world (RW) at least need to navigate their environment and maintain a stable gait. Indeed, eye-movement behavior differs qualitatively, when walking through a natural world as compared to watching the same visual input as videos or series of stills with the head fixed (’t Hart et al., 2009; Foulsham et al., 2011). Moreover, gaze is affected by the difficulty of the terrain to be negotiated (’t Hart and Einhäuser, 2012; Thomas et al., 2020) and critical to guide an individual’s steps (Matthis et al., 2018). Consequently, the constraints and implicit tasks imposed by the environment along with the freedom to move not only the eyes but also the head and the body to allocate gaze, limit the transfer from laboratory studies to real-world settings. At the same time, when aiming for general results beyond a specific application setting – such as sports (e.g., Land and McLeod, 2000; Hayhoe et al., 2012, for a review see Kredel et al., 2017), interface design (Thoma and Dodd, 2019), customer evaluation (Zhang et al., 2018) or driving (Land, 1992; Chapman and Underwood, 1998; Kapitaniak et al., 2015) to name just a few areas where eye-tracking has become a widely used tool – the degree of experimental control in a real-world setting is severely limited. This may become even more crucial when specific tasks such as search shall be studied, rather than free exploration or free viewing. Here, virtual reality (VR) has recently emerged as a viable alternative to overcome the gap between the limited ecological validity of the lab and the limited experimental control of the “wild.”

VR, especially when displayed through head-mounted displays (HMDs) has some intrinsic limitations, such as a restricted field of view or limited resolution. Moreover, physiological factors such as the vergence/accommodation conflict (Kramida, 2016; Iskander et al., 2019), may lead to increased visual stress (Mon-Williams et al., 1998). However, thanks to ever improving display technology, decreasing costs and ease-of-use, over the recent years, VR systems have become a research tool in many fields. This includes – besides the entertainment market – highly regulated fields like medicine [e.g., Dentistry (Huang et al., 2018), education and training (Bernardo, 2017; Izard et al., 2018), simulation, diagnosis and rehabilitation of visual impairments (Baheux et al., 2005; Jones et al., 2020)] and psychotherapy (e.g., autism therapy: Georgescu et al., 2014; Lahiri et al., 2015, fear and anxiety disorders: Hong et al., 2017; Kim et al., 2018; Matthis et al., 2018), as well as in areas directly relevant to psychophysical research such as attentional allocation (Helbing et al., 2020). As fears of long-term negative effects of VR use have so far not been confirmed (e.g., Turnbull and Phillips, 2017), and the recent VR goggles approach photorealistic capabilities while being more and more comfortable to wear, we are now in a position to ask, to what extent a HMD can be used as a proxy for a real-world setting in the context of gaze tracking – a question that has previously only been addressed in a limited scope. Pioneering the use of VR in eye-tracking research, Rothkopf et al. (2007) and Rothkopf and Ballard (2009) demonstrated that with identical visual environments the task – in their case collecting or avoiding obstacles – drastically alters gaze behavior relative to the objects of relevance. Meißner et al. (2017) made use of VR-based gaze tracking in the context of an augmented-reality shopping experience. Anderson et al. (2020) showed that both hand movements and gaze behavior in VR follow the same principles as in real life, at least while watching static natural scenes. VR and gaze tracking are also seeing widespread use in the field of driving simulation, allowing for test scenarios that would be dangerous or difficult to realize in the RW (e.g., Konstantopoulos et al., 2010; Zhang et al., 2017; Swan et al., 2020).

In spite of the increasing use of VR as display technology for eye-tracking experiments, the question as to how faithfully a VR setting mimics real-world constraints with respect to gaze allocation has remained largely unaddressed. Here, we address this issue for walking through a virtual and a real space. For such a direct comparison between gaze allocation when walking through the real and the virtual world, however, participants need to be tested in a sufficiently complex and large environment to allow actual locomotion, which needs to be closely and faithfully matched by a virtual copy of the same environment.

**FIGURE 1:**
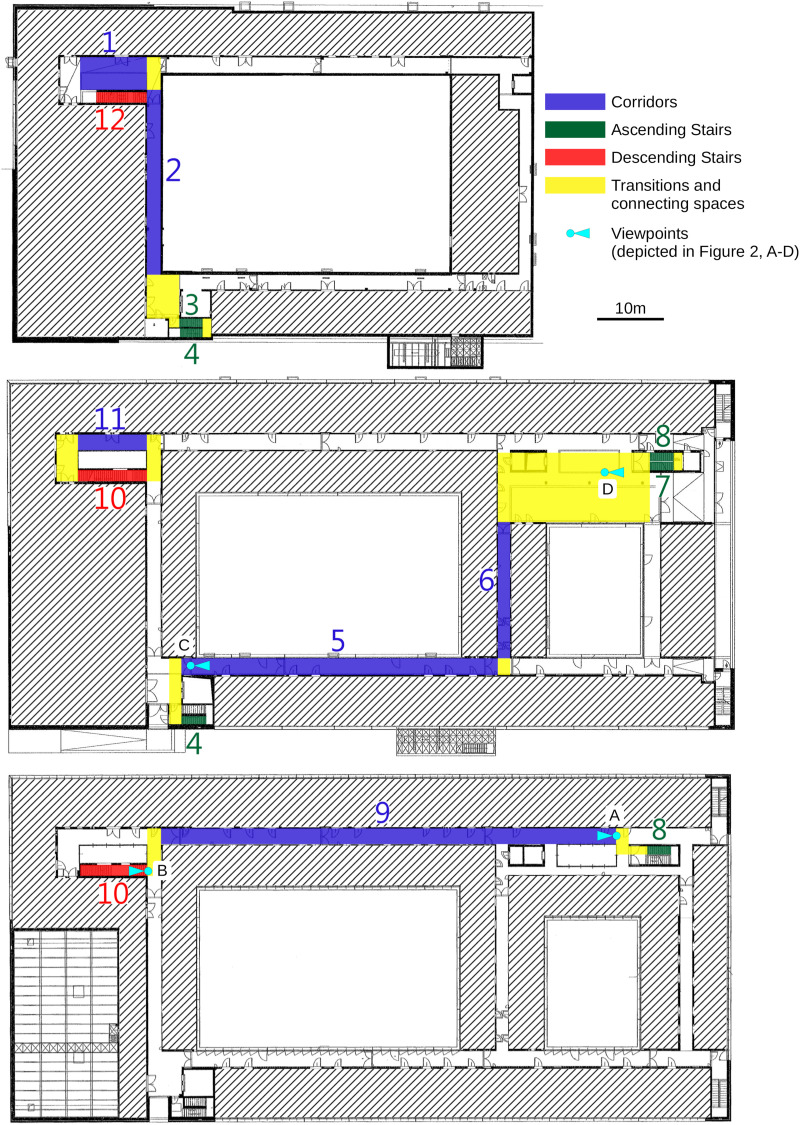
Layout of the three floors (**top panel:** basement, **middle panel:** street level; and **bottom panel:** one floor above street level); of the experimental site and the selected walkway. Different sections are classified by color coding (see legend) and numbered in order of passing. Interior walls and structures irrelevant to the experiment grayed out for data-protection reasons. Map is to scale, scale bar corresponds to 10 m.

In the present study, we compare gaze while walking on a pre-defined path through three storeys of an office building to gaze while moving on a virtual high-fidelity copy of the same path (Figure 1 and sample videos in the Supplementary Material). In VR, participants control their translational movement by a handheld controller (and do not actually translate), while they do execute rotational movements that are transformed into the matching rotational movement in the VR. We predefine different zones on the path (factor “sector” with levels “corridors,” “ascending stairs,” and “descending stairs”) and assess robust measures of eye-movement behavior for both the RW and the VR (factor “world” with levels “VR” and “RW”). Assuming that the movement in VR is a good proxy for locomotion in the RW (with respect to gaze measurements), we would expect that differences in these measures found in the RW are also found in the VR, and remain largely unaffected by the choice of world. That is, under the hypothesis that VR faithfully approximates the RW, we expect main effects of the factor sector, but no interaction between sector and world for dependent variables characterizing relevant aspects of gaze allocation.

## Materials and Methods

### Comparing Different Worlds

To compare gaze behavior between VR and RW, it is desirable to expose the participants to VR-generated surroundings that are as closely matched to the RW surroundings as possible. For practical reasons, the Physics building of Chemnitz University of Technology was chosen as the real-world location for this study and also modeled in VR.

### Real World

**FIGURE 2:**
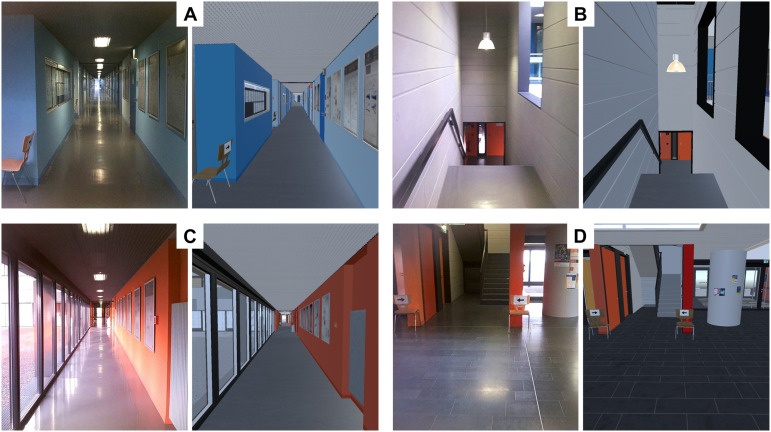
Comparison of simulation and reality. Pairs of corresponding sample frames taken from the real world **(left)** and the virtual reality **(right)** recordings. VR rendering quality settings were as used during the experiment, (image quality can appear lower in the article pdf due to compression) RW were cut from the SMI glasses scene-camera recordings. **(A)** A corridor without windows. **(B)** Descending staircase. **(C)** A corridor with windows. **(D)** Parts of the lobby (not used in the analysis) and the entrance to an ascending staircase.

Participants were instructed to walk through the building on a pre-defined route (Figure 1) at “their usual walking speed without unnecessary stopping.” To enable participants to follow the route without actively engaging them at every turn, landmarks were placed at critical spots pointing in the correct direction. To avoid making the landmarks overly salient by falling out of the building context, a type of office chair abundantly available throughout the building was chosen. A plain white A4-sized paper with a black printed arrow was attached to the backrest, pointing the way (Figure 2).

The route started in the basement in the laboratory’s commons area, and went through several corridors, lobbies and staircases until it returned on a different route to the same commons area. A detailed route description can be seen from Figure 1, including the segmentation into types of sectors (terrain) for the analysis. The route was inspected before each session, unforeseen obstacles were removed and any doors possibly interfering with the route were blocked open, such that participants did not have to interact with any object in their path. At the end of each session, the route was inspected again, and each recorded scene cam video was also manually inspected for such anomalies.

The experiment was conducted in the early evening hours of the European summer (ca. 18–21 h CEST), as these hours afforded both good natural illumination and minimal traffic within the building. Nonetheless, on occasion there were unforeseen obstacles in the path of the participant, and in 10 instances (max. 1 per individual) participants encountered other persons or doors not part of the walking route left open during the trial. Even though participants reported to believe that those incidents were part of the experiment, the corresponding periods during which the disturbance persisted (i. e., was visible) were excluded from gaze-data analysis to avoid data contamination.

### Virtual Reality

To achieve best possible comparability between VR and reality, a high-fidelity 3D model of the entire physics building was developed, allowing identical walking routes in VR as well as reality (for a sample screenshot comparing RW and VR, see Figure 2; sample movies depicting both virtual and RW are available as Supplementary Material^1^). No human-like characters or avatars were placed in the virtual scenario. The software packages Blender (v. 2.79) and Unity (v. 2018.3.0f2) were used for developing and rendering the VR model. The internal details of the building were represented in the VR with great attention to detail and quality, including physical objects such as door handles, fire extinguishers, air vents, plants, and readable posters and showcases with objects inside. As a result, the virtual environment consists of 1,607 objects, whose total polygon number adds up to 4,453,370. Three hundred and forty different materials were used to texturize these objects. To limit the hardware load caused by the high polygon count of the model, a combination of culling operations offered by the Unity engine were used to minimize drawing operations without compromising visual quality. The main components of the model are the publicly accessible areas (corridors, staircases) of the reference building. They extend over five floors, which are connected by 242 steps and a virtual elevator that stops on four floors (elevator was not used in the present experiment). There are more than 200 doors and a similar number of scientific posters located along the corridors. Besides the public areas of the building, the laboratory where the virtual part of the experiment took place was also modeled. It served as starting and ending point of the predefined path in the experiment and made it easier for the test persons to switch between the RW and the virtual environment, because they started (finished) in the same position in VR where they put on (off) the headset in the RW. One seminar room and one office also were modeled in their entirety – for demonstration purposes and for use in follow-up experiments.

#### Matching Simulation and Reality

Within the VR, the height of origin of the participant’s field of view (i.e., the virtual camera position) was adjusted individually to the physical height of each participant, to optimally match the visual appearances of the virtual and the RW. Proper camera height is also helpful to assist participants in fully immersing themselves in the VR, including the perceived ownership of their virtual bodies (van der Veer et al., 2019). The route that participants were to take during the experiment was marked with virtual copies of the marker chairs described above. Every chair’s location and orientation from the real-world trials were copied faithfully to the VR, including the attached paper with the printed arrows (Figure 2).

#### Navigation in VR

Participants viewed the virtual building from a first-person perspective while moving their virtual body (“avatar”) through the building. To avoid cyber sickness (or motion sickness, Golding and Gresty, 2005; Mazloumi Gavgani et al., 2018) while maintaining a naturalistic mode of navigation, a hybrid between physical tracking and joypad navigation was implemented. Forward (and if needed, backward) movement was controlled by means of the joypad on top of a VR controller. Rotational movements, in contrast, were actually executed by the participant and transformed to VR by tracking the VR controller held close to the body. When a participant pressed forward, the avatar would accelerate smoothly to a top speed of 1 m/s (equivalent) in the direction the participant’s body was facing. Participants were instructed to rotate their whole body (and thus the VR controller with it) to determine the orientation of their avatar in the VR world. Through this, head movements were independent of motion direction, allowing for natural viewing behavior while at the same time allowing for an intuitive, semi-naturalistic navigation through the VR space. The top speed of 1 m/s was chosen to minimize the probability of motion/simulator sickness during the course of the experiment. The overall walking time was also relatively short (expected < 10 min), which should also help to avoid cyber sickness during the course of the experiment (Dużmańska et al., 2018; Mazloumi Gavgani et al., 2018). Indeed, no cases of cyber sickness were reported by the participants.

### Experimental Setup and Gaze Recording

#### Real World

Real-world eye tracking was performed with a wearable eye tracker manufactured by SensoMotoric Instruments (SMI Eyetracking Glasses, ETG 2.1). Gaze data and scene camera video were recorded with a specially modified cell phone (Samsung Galaxy S5), which participants carried in a belt pocket. Gaze data were initially recorded at 60 Hz, while scene video was recorded at 25 Hz at a manufacturer-defined field of view of 60° horizontally and 46° vertically, which corresponds to the gaze tracking range of the device. The manufacturer’s built-in calibration was used to achieve a correct mapping between gaze and scene video. Calibration markers (3 × 3, spaced at 10° horizontally and vertically) were attached to a wall, and participants were instructed to fixate each marker for at least 2 s, once before walking along the path for manual inspection of the calibration prior to recording as well as once at the end of the experiment to allow for drift estimation.

#### Virtual Reality

For VR presentation and interaction, an HTC Vive VR headset was used in combination with a Vive hand controller. The headset offers a stereo display with a physical resolution of 1,080 × 1,200 pixels per eye at a refresh rate of 90 Hz, and a field of view of approx. 100° horizontally and 110° vertically. Position and rotation of the headset in space is tracked by means of 2 “Lighthouses” (laser scanners). While the VR and tracking capabilities of the headset were unchanged from the standard commercial package, eye tracking was realized through an aftermarket add-on manufactured by Pupil-Labs (Pupil Labs GmbH, Berlin, using Pupil Capture v. 1.11-4), consisting of two infrared cameras mounted inside the head set, tracking one eye each with a nominal frequency of 120 Hz at a camera resolution of 640 × 480 pixels. Eye tracking and VR computations were performed on a laptop (ASUS GL502VS, Intel Core i7 6700HQ, Nvidia GeForce GTX1070 GPU), allowing for time-synchronized data recording of both VR and eye/gaze events. The cables leading to the VR headset were loosely suspended from the ceiling above the participant to avoid exerting forces on the participants’ head and neck.

To achieve a correct mapping between measured pupil position and gaze position in VR, the built-in calibration routine of the Pupil Labs eye tracker was followed by a custom calibration sequence, consisting of 3 × 3 calibration markers positioned at a grid spacing of 10° vertically and 11.5° horizontally. Participants were cued which marker to fixate by a change in marker color, and were requested to maintain fixation until the next marker was highlighted in random order by the operator (at least 3 s of fixation time each). The recorded raw data was then projected onto the known positions of the calibration grid using a 2-dimensional polynomial fitting procedure (Drewes et al., 2014). At the end of the VR recording session, the procedure was repeated to allow for drift evaluation.

### Procedure and Participants

The order of conditions (VR vs. RW) was balanced across participants. Including briefing, data collection and debriefing, the experiment lasted about 40 min, depending on individual walking speed. Twelve individuals participated in the experiment (9 women, 2 men, and 1 unreported) with an average age of 22.8 years (18–33). Visual acuity was tested by means of a Snellen chart; all participants reported to be healthy and being able to walk and climb stairs without any restrictions or aid. Participants were explicitly instructed prior to the experiment that they should abort the experiment, if they experienced any motion sickness or discomfort; when asked informally in debriefing, no one indicated any signs of either motion sickness or discomfort. Participants were remunerated for their participation by 6€/h or course credit.

All procedures were performed in accordance with the Declaration of Helsinki, and were evaluated by the applicable ethics board (*Ethikkommission der Fakultät HSW, TU Chemnitz*) who ruled that no in-depth evaluation was necessary (case-no.: V-274-PHKP-WET-Augenb-11062018).

### Data Processing and Analysis

For eye movements recorded in VR, as a first step the calibration solution as described above was applied. Three participants had to be excluded, as data quality did not allow for proper calibration. For one additional participant, data recording failed due to technical issues. For the remaining eight participants, those samples were marked invalid where the corresponding pupil size was zero, as this indicated no visible pupil; for example, due to lid closure or because the pupil was outside the tracking area. For eye movements recorded in the RW, no additional calibration was required, and no further participants had to be excluded. Gaze data is generally expressed in calibrated degree visual field, with increasing values from top (gaze up) to bottom (gaze down) and left to right.

In order to relate gaze patterns with different sections of the route through the building, the continuous gaze data for both RW and VR were cut into segments according to the location of the participant along the walking route at a given time. Three different types of segments were identified for the analysis: straight walkways (“corridors”), staircases leading up (“ascending stairs”) and staircases leading down (“descending stairs”). In the selected routing, the staircases leading up are interrupted by a platform with an about-turn in the middle between two floors, resulting in two stair segments per floor, whereas the descending staircases lead straight through to the next lower floor. Connecting areas and areas that could not be classified as one of the three sector types were excluded from the analysis (e. g., the turns between corridors, and a large lobby). In total, there were 6 corridors, 4 ascending stair segments and 2 descending stair segments, covering a walkway length of approximately 285 meters.

Generally, the demands of navigating the RW compared to the VR may differ, even in the most sophisticated VR model. As one possible marker of such differences, we analyzed the amount of time spent attending navigational aids, i.e., the duration the chairs with directional arrows placed among the walking route were looked at. This required us to determine the position of the chairs in the participants’ field of view for each recorded frame. While in principle there exist methods in VR to conveniently identify objects hit by the observer’s gaze (e.g., ray casting, Watson et al., 2019), these methods cannot be applied in the RW. To achieve comparability between the data generated from the VR and RW recordings, we chose the following method: for the real-world scene videos, we trained a deep learning algorithm (Mathis et al., 2018; Nath et al., 2019) to recognize the chairs. The algorithm delivers the 4 corner coordinates of the most likely position of a chair in each frame, together with a confidence value for its estimate. Visual inspection revealed that those positions with confidence values above 0.9 (on a scale from 0 to 1) indeed reliably identified chair positions; positions with a confidence value below this threshold were discarded.

In VR, locating chair positions was realized by re-rendering each frame for each participant from the recorded coordinates, such that all pixels in the frame were black, except for the chairs, which were rendered blue. The identified blue pixels were then fitted with a trapezoidal shape, resulting in the 4 corner coordinates of each chair, comparable to the data obtained from the RW scene videos.

In both worlds, the distance of the current gaze from the nearest pixel contained in the chair trapezoid was then computed, and samples were considered to be on a chair whenever the distance was no larger than 1 degree. As the tracking range of the VR system is much larger than that of the RW system, chairs may be visible at distances further from the current gaze point than the maximum of the RW system. This might exaggerate the average gaze-to-chair distance in the VR world. To avoid this, the VR analysis was limited to those frames where both chair and gaze were within the corresponding tracking range of the RW system.

To visualize gaze distribution patterns in both RW and VR, heat maps were computed from gaze position data. Sample data was accumulated in 2D-histograms with a bin size of 1°, spanning a range of ±50°. Data outside this range was accumulated in the outermost bins. For display purposes, histograms were then normalized to a common range for each participant, and smoothed with a Gaussian low-pass filter (FWHH radius of 1 bin). To accommodate zero values on the logarithmic plotting scale, a regularization (+ 5% of the scale) was performed on all histograms.

The RW headset does not feature sensors for head movement recording; in VR, however, these sensors are integrated in the headset functionality as they are essential for the automatic updating of the virtual perspective. The zero-point of the headset orientation depends on the precise way in which the headset is positioned on each individual participant; we therefore chose the average position of the headset during the corridor sectors as the zero reference to allow for a comparison of head position data between sectors within the VR.

In order to improve gaze comparison between devices, we chose the visual horizon as a common point of reference for some analysis (eye-in-world). In the VR system, the horizon as well as the head angle relative to the horizon can readily be tracked. In the RW setting, however, the eye tracker used does not offer head tracking capability, and the position of the horizon in the visual field is not known. The horizon in the recorded scene video was therefore tracked by a hybrid between manual marking and a correlation-based tracking algorithm (utilizing MATLAB’s xcorr2 function). Every nine frames, the horizon was marked manually in the current video frame, and the marked position was used as a reference point for the correlation tracker, which then provided the movement of the reference point for the both the following and the previous 4 frames as output. This approach for the RW scenario requires a clearly visible and identifiable horizon, at a far enough distance such that the different physical heights of the participants would not affect the angle of view at which the horizon was found in the image. One long corridor (section number 5 in Figure 1) with a large window at the end allowed for a reliable tracking of the far horizon and was thus chosen as the reference sector for this analysis. The vertical gaze position while passing through this sector was then subtracted from the position of the horizon on a frame-by-frame basis to achieve “eye-in-world” coordinates.

Histograms of eye-movement directions were created to profile general eye movement behavior. For each sample, the difference in gaze position relative to the previous sample was computed. Non-zero differences were then binned by direction of gaze movement, in bins of 45°, centered on the cardinal and oblique axes (resulting in a total of eight bins: [−22.5° 22.5°], [22.5° 67.5°], [67. 5° 112.5°], [112.5° 157.5°], [157.5° 202.5°], [202.5° 247.5°], [247.5° 292.5°], [292.5° 337.5°]). Histogram data was then normalized per individual to unit integral before averaging across participants.

Gaze velocity histograms were computed from gaze velocity values as defined by the absolute position difference between two neighboring valid gaze samples, normalized by the sample time difference. Gaze samples without valid neighbors were excluded from analysis. Samples were then accounted for in logarithmically spaced bins (in octaves, i.e., < 1°/s, 1–2°/s, 2–4°/s, 4–8°/s, 8–16°/s, 16–32°/s, 32–64°/s, 64–128°/s, 128–256°/s, 256–512°/s, > 512°/s).

We computed saccade rate (number of saccades per second) for each participant, in both RW and VR, separately for each sector, according to the method proposed by Engbert and Kliegl (2003), manually adjusting their algorithm’s noise threshold (Lambda) individually for each participant and world.

Data was analyzed in GNU Octave (v4.4.1 and v5.2.0, Eaton et al., 2020), MATLAB (MATLAB R2019b, 2019), and R (v3.6.1, R Core Team, 2020). Repeated measures ANOVAs with factors “world” and “sector” were performed using the ezANOVA function in R (Lawrence, 2016), and Greenhouse-Geisser corrected *p*-values are reported along with uncorrected degrees of freedom and the Greenhouse-Geisser ε (ε_GG_) when Mauchly’s test indicated a significant violation of sphericity at a 5% level. Kolmogorov-Smirnov tests did not indicate any deviation from normality, although the sensitivity of this test (and any test for normality) is limited by the comparably low sample size.

## Results

For the included 8 participants (see section “Materials and Methods”) data quality in the VR condition was in general better for the left than for the right eye. The left-eye data of the VR condition was thus chosen for further analysis. Sample data was mapped to degree visual field as described in the “Materials and Methods” section.

**FIGURE 3:**
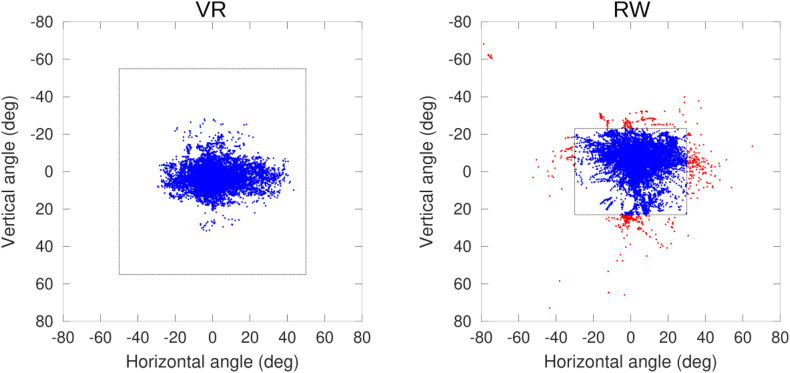
2D-Distribution of calibrated gaze position in head-centered coordinates, one sample participant shown. Dashed boxes indicate the manufacturer-specified tracking limits (Pupil Labs/VR: 100 × 110°, SMI/RW: 60 × 46°), red data points are outside the specified tracking limits, but the side (left/right and up/down) relative to the limits is still well-defined.

The median rendering frame rate of the VR was 73 Hz, with 95% of all frames rendered at 35 Hz or faster (this lower 5% percentile varied between 32 and 40 Hz across participants). The gaze sampling frequency of the VR tracker measured 119.1 Hz (120 Hz nominal) for 96% of all samples, with a minimum of 94% and a maximum of 99% across participants. For the VR condition, we on average recorded 400 s (SD 46 s) of data per participant, amounting to a total of 355718 data points. Of those, 99.5% (SD 1.2%) were valid samples. Of those, all fell within the tracking window specified by the manufacturer (110° vertically and 100° horizontally, Figure 3). In the RW, we recorded 333 s (SD 17 s) of data, amounting to a total of 160220 data points, of which 85.5% (SD 6.6%) were valid samples, falling within the range (60° horizontally, 46° vertically) for which the manufacturer specifies tracking quality (Figure 3B). However, it is still reliable in which direction they are outside the tracked range (left/right and up/down). We therefore included data points outside the manufacturer-defined range in the computation of the median position and inter-quartile ranges, where their exact position does not influence these measures (given that no more than 50% of data fall outside on one side).

**FIGURE 4:**
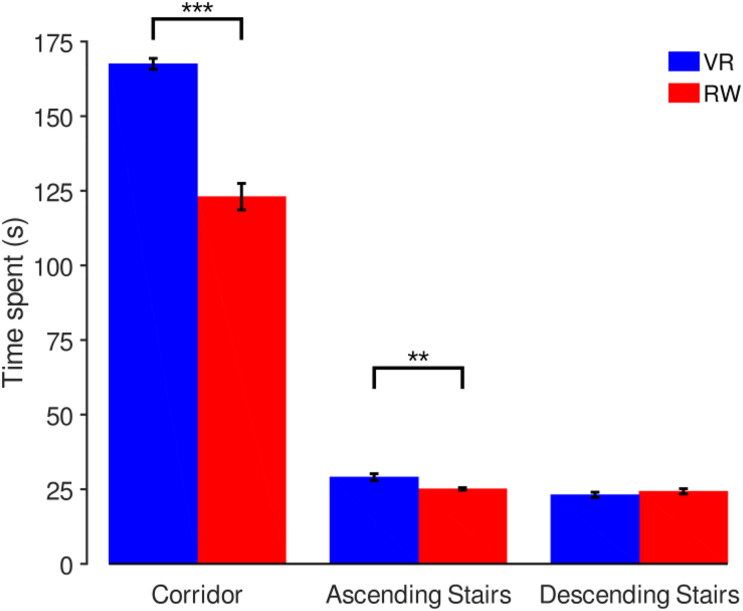
Comparison of time spent in different sectors. Mean and SEM across participants, with significant differences indicated above (***p < 0.001, **p < 0.01).

Real world and VR parts of the experiment did not generally last the same time (see above, paired *t*-test, *t*(7) = −3.91, *p* = 0.006, including the entire walking route). However, the order of the path segments was always the same as the routing through the real and virtual buildings was identical. Pairwise tests show that time spent differs significantly in the “Corridor” sectors [means: VR 167.5 s (SD 12.6), RW 123.0 s (SD 12.6), *t*(7) = −8.92, *p* < 0.001] and the “Ascending Stairs” sectors [VR 29.1 s (SD 3.2 s), RW 25.1 s (SD 1.2), *t*(7) = −3.54, *p* = 0.009], but not in the “Descending Stairs” sectors [VR 23.13 s (SD 2.5), RW 24.3 s (SD 2.5), *t*(7) = 0.82, *p* = 0.439]. In summary, participants were slower in VR for corridors and ascending stairs, but not descending stairs (Figure 4).

At the end of the measurement in each world, we estimated the calibration error using the same grid as used for calibration at the start for validation. This analysis revealed substantial amounts of drift (VR: 6.4° ± 5.7°, RW: 10.8° ± 2.6°) over the course of the recording, but no significant bias in drift direction for neither the VR [mean and SD, horizontal: 0.9° ± 2.0°, *t*(7) = 1.35, *p* = 0.219; vertical: −0.5° ± 8.6, *t*(7) = −0.15, *p* = 0.885] or the RW [horizontal: 0.1° ± 1.3°, *t*(7) = 0.28, *p* = 0.791; vertical: 3.3° ± 6.0°, *t*(7) = 1.55, *p* = 0.166]. No significant differences were found for the drift direction biases between VR and RW [horizontal: *t*(7) = −1.15, *p* = 0.285; vertical: *t*(7) = −0.89, *p* = 0.402]. Qualitative inspection of the data showed that within each individual all nine validation points are offset by about the same direction and magnitude, indicating that the main source of error was indeed drift, which likely resulted from movement of the headset relative to the head. Importantly, this implies that measures that are not based on absolute position – such as spread [inter-quartile-range (IQR)] and velocity – remained unaffected by this measurement error.

### Gaze Distribution (Eye-in-Head)

**FIGURE 5:**
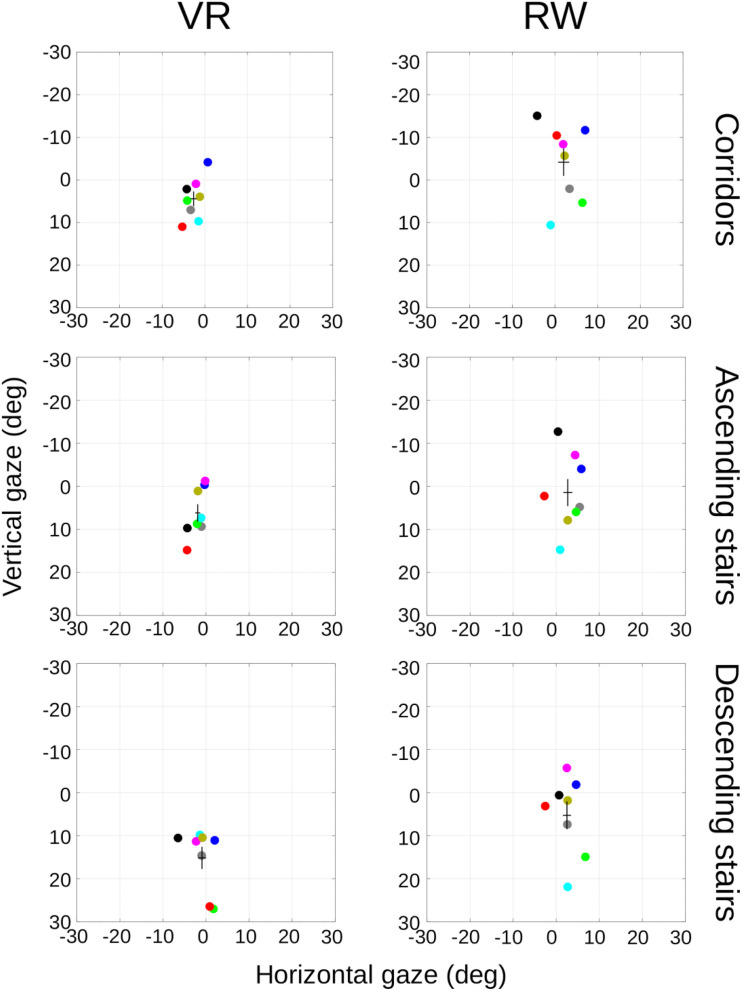
Median of calibrated gaze positions in head-centered coordinates, separated by sector and world. Individual colored dots represent individual participants (matched across panels), black crosses represent mean and SEM across participants.

Average eye-in-head orientation was computed separately for the three different sector types. Per participant, we characterized the gaze distribution by its median in the horizontal and vertical dimensions (Figure 5). Repeated measures ANOVAs with factors world (levels: VR and RW) and sector (levels: corridor, ascending stairs, and descending stairs) revealed significant main effects of vertical gaze direction for both the factor world (VR vs. RW, positive values represent downward gaze; mean of medians across participants and standard deviation: 8.59° ± 7.52° vs. 0.86° ± 9.51°, *F*(1,7) = 7.34, *p* = 0.030) and the factor sector (*F*(2,14) = 33.61, *p* < 0.001), but no significant interaction (*F*(2,14) = 1.77, *p* = 0.206). Follow-up paired *t*-tests (Table 1) show all sectors to differ from each other [corridor vs. ascending stairs, *t*(7) = −3.60, *p* = 0.009; corridor vs. descending stairs, *t*(7) = −6.97, *p* < 0.001; ascending vs. descending stairs, *t*(7) = −5.21, *p* = 0.001]. A significant main effect for the factor world was also found for horizontal gaze, although numerically the absolute difference was much smaller [−1.82° ± 2.18° vs. 2.40° ± 3.04°, *F*(1,7) = 38.8, *p* < 0.001; positive values represent rightward gaze direction]. There was no significant main effect for the factor sector on the horizontal gaze direction [*F*(2,14) = 2.11, *p* = 0.158, see Table 1], and no interaction [*F*(2,14) = 0.56, *p* = 0.491, ε_GG_ = 0.54]. These data show that the sector significantly influences gaze behavior; importantly, the lack of a significant interaction indicates that this influence is independent of whether the terrain is actually negotiated in the RW or just virtually in VR. The main effect of world in the vertical direction is somewhat surprising (if anything, one would have predicted a lower gaze in the RW). This may however be influenced by differences between the gaze recording devices or posture-related differences, as such systematic offsets are unavoidable when considering eye-in-head position data (see section “Eye-in-World…” below).

**TABLE 1:** Comparison of average gaze between sectors.

Sector	Coordinate	VR	RW	*t*-score	*p*-value
Corridor	X	−2.62° ± 1.97°	2.4° ± 3.04°		
	Y	4.43° ± 4.91°	−4.15° ± 9.11°	3.11	0.017*
Ascending stairs	X	−1.92° ± 1.64°	2.69° ± 2.98°		
	Y	6.17° ± 5.71°	1.46° ± 8.92°	1.37	0.212
Descending stairs	X	−0.93° ± 2.73°	2.49° ± 2.72°		
	Y	15.16° ± 7.28°	5.27° ± 9.13°	2.73	0.029*

*Mean of medians and standard deviation across participants, as well as follow-up statistics (paired t-test, VR vs. RW, df = 7) for the Y-coordinate where the factor sector had a significant main effect. Significant values (p < 0.05) are marked by an asterisk.*

**FIGURE 6:**
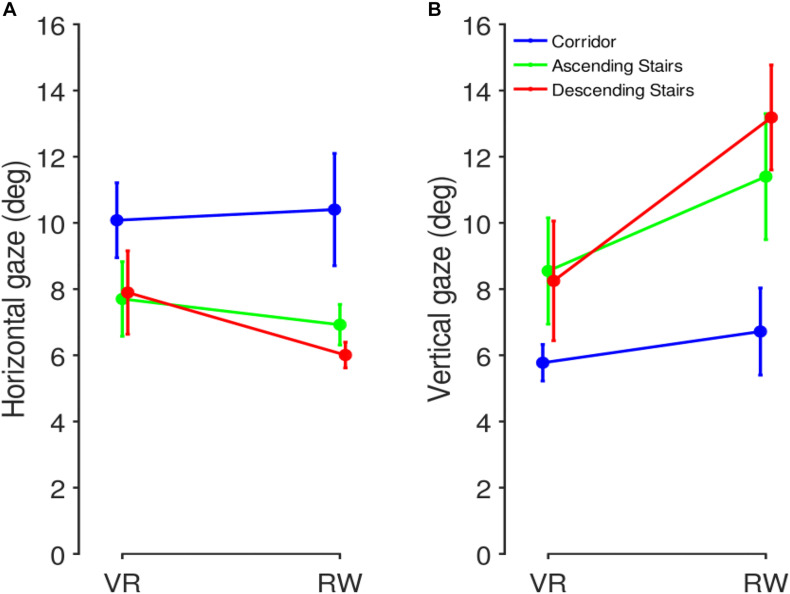
Inter-quartile-range (IQR) comparison between RW and VR. IQRs were computed as a measure of gaze spread, separately for the three sector types. **(A)** Horizontal gaze IQR. **(B)** Vertical gaze IQR. Mean and SEM across participants.

While the median location is a measure that is robust to outliers, in particular against points falling outside the manufacturer-specified tracking range, it is susceptible to systematic offsets and does not capture the overall distribution of the data. Consequently, we also considered a measure of spread in the horizontal and vertical dimension. The IQR is robust to both outliers (as long as outliers constitute less than 25% on either side) and offsets and thus well suited as an additional means to describe the data at hand. We computed the IQR for each participant and sector (Figure 6). In the horizontal direction, we found a significant main effect for the factor sector [*F*(2,14) = 7.12, *p* = 0.007], but not the factor world [*F*(1,7) = 0.75, *p* = 0.415] with no significant interaction [*F*(2,14) = 0.95, *p* = 0.410]. Similarly, in the vertical direction, we found a significant main effect for the factor sector [*F*(2,14) = 9.69, *p* = 0.002], but not the factor world [*F*(1,7) = 3.23, *p* = 0.115] with again no significant interaction [*F*(2,14) = 2.68, *p* = 0.104]. This corroborates the findings of the median position data: gaze distributions are influenced by the sector and this influence does not depend on whether the locomotion takes place in the RW or is simulated in VR.

**FIGURE 7:**
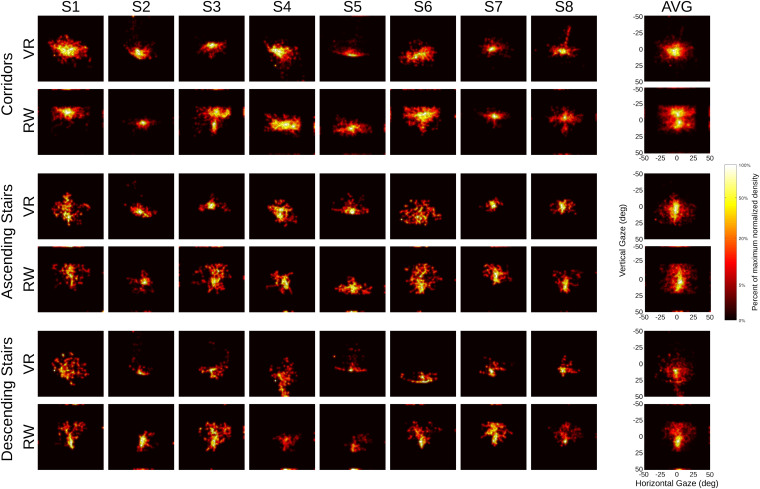
Heat maps of gaze distribution, separated by world (virtual reality vs. real world) and sector type. Data normalized for each participant (S1 … S8) and displayed with a logarithmic scale (with a slight regularization to accommodate zero values), see color bar. Average data across participants in column (AVG). Outer margin in each panel represents data points outside of the specified tracking area, squares span ±50° in each dimension.

To illustrate the individual gaze patterns visually, heat maps were generated from normalized 2D-histograms (Figure 7). By visual inspection, gaze patterns show substantial inter-individual differences, ranging from a narrow, focused appearance (e.g., S2) to a wide-spread pattern (e.g., S6). Participants differ both in horizontal and vertical spread. For the average of the corridor condition in the RW, an apparent two-peak pattern emerges, which is not apparent in the VR condition. Visual inspection reveals this to be due to different peak locations across individual participants rather than within. Some of the resulting distribution patterns (e.g., participant S3, Figure 7) resemble the T-shape previously reported in natural gaze behavior (Calow and Lappe, 2008; ’t Hart et al., 2009). The T-shaped pattern is thought to represent gaze behavior during navigation, the T-trunk resulting from gaze directed toward the terrain immediately ahead, perhaps to verify navigability, and the T-bar representing gaze directed further up and looking toward the sides, perhaps to register the surroundings or to plan further ahead. To identify possible differences in this T-shaped gaze allocation between the different worlds, we quantified the degree of T-shaped gaze distribution in each participant: the gaze data was split at the vertical median, leaving an upper and a lower half containing equally many data points. For each of these halves, the horizontal IQR was then computed, and the result of the upper half was divided by the result of the lower half. The resulting ratio was then used as input to an ANOVA with factors world and sector, as above. The difference in IQR ratios was significant only for the factor sector [*F*(2,14) = 14,51, *p* < 0.001], but not the factor world [*F*(1,7) = 3.28, *p* = 0.110] and there was no significant interaction [*F*(2,14) = 3.29, *p* = 0.068]. As there was a trend to an interaction, we decided to analyze these data separately by sector. While the ratios averaged across participants were almost identical for corridors (VR: 1.16 SD 0.32; RW: 1.15 SD 0.40), the ratios on the stairs were larger in the RW, suggesting a more pronounced T-shape (ascending, VR: 1.27 SD 0.38; RW 1.58 SD 0.48; descending, VR: 1.54 SD 0.74; RW, 2.77, SD 1.42). This is an indication that for specific terrains where information from the ground is particularly relevant for foot placement (as the stairs in our case), subtle differences between VR and RW may start to emerge.

### Eye-in-World: Relating Eye-in-Head Coordinates to the Horizon

The results reported so far are in head-centered (eye in head) coordinates, where position data as such may include offsets due to the different eye trackers used in the RW and VR condition. To compensate for this effect and to estimate gaze relative to the world, we computed eye-in-world coordinates by referencing gaze orientation relative to the horizon (see “Materials and Methods”). For the RW, this analysis requires the horizon to be identifiable, but at greater distance. Hence, we restricted this analysis to one corridor, where the horizon was visible through a window at the end of the hallway. No other sector shared this property, making this analysis feasible only for the chosen corridor.

**FIGURE 8:**
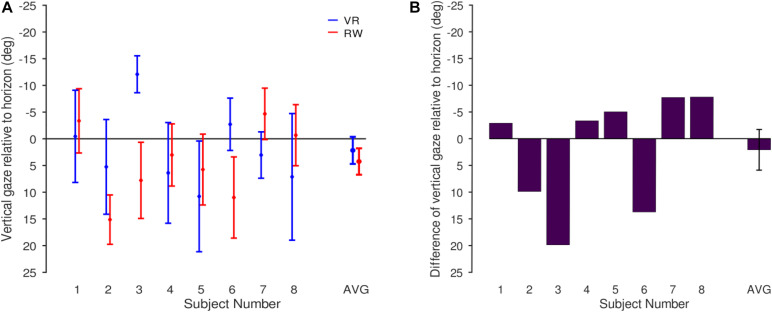
Gaze distribution relative to the horizon for the corridor for which head-in-world direction was determined (the one depicted in Figure 2C, see text). **(A)** Individual participants (mean and SD) and averaged across participants (mean and SEM). **(B)** Difference between VR and RW (VR-RW) for each participant and averaged across participants (mean and SEM).

On average, gaze in the VR condition was 2.2° below the horizon (SD = 7.2°) and 4.2° in the RW condition (SD = 7.0°). This difference was not significant [paired *t*-test, *t*(7) = 0.55, *p* = 0.600; Figure 8]. In sum, contrary to the eye-in-head data, we found no evidence for systematic differences for eye-in-world position. This makes it likely that the observed difference for eye-in-head coordinates, for which no absolute straight-ahead reference is available in VR, is primarily a consequence of headset placement relative to the participants’ head. Importantly, all relative measures – spread and velocity – are insensitive to this placement as well as to its possible drift over the course of the experiment.

### Head Movements

As head movement data was generally not available for the RW, we analyzed head-in-world movements in detail only for the VR. Horizontal orientation (heading) of the headset depended strongly on the position along the walking route. This stemmed on the one hand from different sectors having different compass alignments (lead heading); on the other hand, at each transition from one sector to the next, participants were physically required to turn. Due to the rectangular layout of the building, the angle of the change in route direction most often measured 90°, although the turn between segments of the upward stairs measured 180° (see Figure 1). Most sectors therefore start and end with a turn of at least 90°, which lead participants to make anticipating head movements in the direction of the turn as they approached the end of each sector. As the length of the individual sector types differs strongly (see Figures 1, 4), head movements in the horizontal direction (yaw) for each sector type are thus contaminated to different degrees with the initiation and termination of the turns executed by the participants. We therefore limited our analysis to vertical (pitch) and roll head movements, analyzing both the mean angle and the IQR of the angular distribution in an ANOVA with the factor sector only. Average vertical head position relative to the corridors was downward 0.55° ± 2.77° for ascending stairs and downward 14.6° ± 3.64° for descending stairs; average roll relative to corridors was 0.34° ± 1.04° to the right for ascending stairs and 0.27° ± 2.08° to the left for descending stairs. We found a significant effect on average vertical head position [*F*(2,14) = 86.49, *p* < 0.001], but not on roll [*F*(2,14) = 0.40, *p* = 0.681], with no significant effect on the IQR for either vertical position [*F*(2,14) = 2.86, *p* = 0.091] or roll [*F*(2,14) = 0.61, *p* = 0.559].

### Angular Distribution of Gaze Direction Changes

**FIGURE 9:**
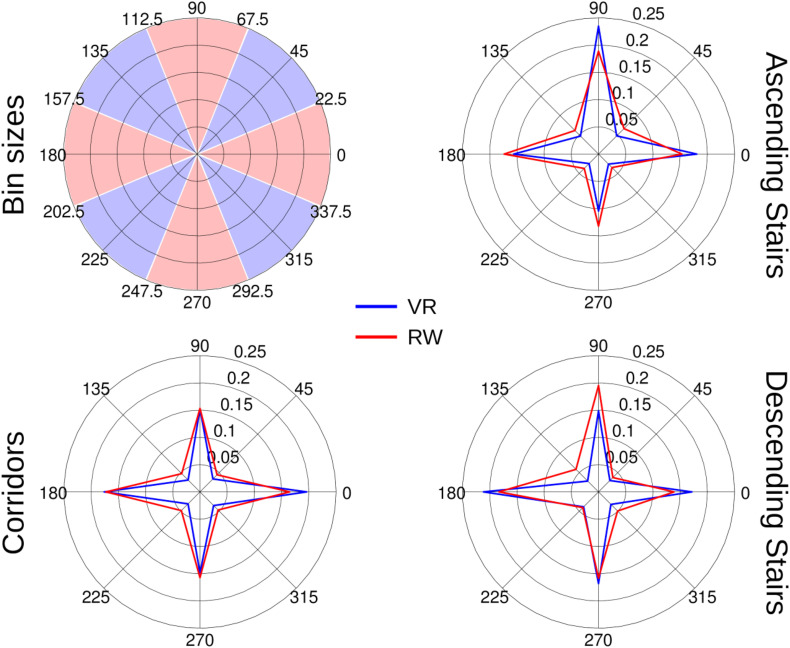
Histograms of eye-movement directions as polar plots. Non-zero differences in gaze position between consecutive samples were accounted by direction of gaze movement in bins of 45° centered on the cardinal and oblique axes (see top–left panel). Data was normalized per individual to unit integral before averaging across participants. Directions as shown correspond to directions of gaze movements (0° corresponds to rightward, 90° to upward gaze movements, etc.).

Eye movements during free, explorative behavior are generally highly variable. Differences in this behavior may signify differences in the processing of the visual environment. To profile these eye movements and identify possible differences between RW and VR, we assessed the directional distribution of gaze differences between recorded samples as well as the corresponding distribution of absolute gaze velocities. Similar to previous research (Einhäuser et al., 2007; Meißner et al., 2017), we find cardinal directions (horizontal/vertical) more abundant than oblique directions (Figure 9). To quantify this difference, we computed the ratio between the sum of the fraction of movements in cardinal directions (here defined as 45° wedges around the cardinal axes, Figure 9) and oblique directions and used it as input to an ANOVA with the factors world and sector. We found a significant effect for the factors world [*F*(1,7) = 40.31, *p* < 0.001] and sector [*F*(2,14) = 5.36, *p* = 0.047, ε_GG_ = 0.56], without significant interaction [*F*(2,14) = 0.36, *p* = 0.706].

### Distribution of Gaze Velocities

**FIGURE 10:**
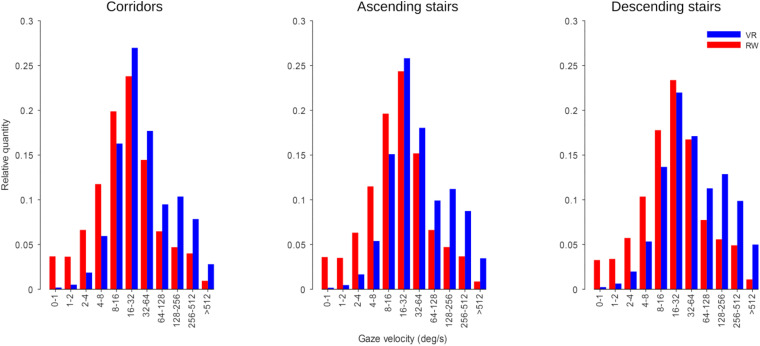
Gaze velocity histograms. Velocity bins are log-spaced (in octaves) and labeled by the velocity contained each bin.

Gaze velocities were computed and averaged across participants (Figure 10). The velocity distributions for RW and VR are similar in that they peak between 16 and 64°/s, but differ in that the RW measurements contained more slow velocities (<16°/s) and the VR measurements contained more fast velocities (>128°/s).

We computed an ANOVA on the per-participant medians of the velocities, with factors world and sector. We found a significant main effect for the factor world [*F*(1,7) = 7.92, *p* = 0.026], but not the factor sector [*F*(2,14) = 1.89, *p* = 0.209, ε_GG_ = 0.55], without significant interaction [*F*(2,14) = 1.02, *p* = 0.352, ε_GG_ = 0.55]. When separating this analysis by horizontal and vertical gaze component, for the horizontal component we find a significant effect for the factor world [*F*(1,7) = 16.94, *p* < 0.001] but not the factor sector [*F*(2,14) = 2.87, *p* = 0.124, ε_GG_ = 0.60], again with no significant interaction [*F*(2,14) = 1.92, *p* = 0.184]; We found no significant main effect or interaction for the vertical component [world: *F*(1,7) = 3.26, *p* = 0.114; sector: *F*(2,14) = 1.76, *p* = 0.226, ε_GG_ = 0.51; interaction: *F*(2,14) = 0.47, *p* = 0.52, ε_GG_ = 0.51].

### Comparison of Saccade Frequency

The mean saccade rates were computed for each participant: 2.03 ± 0.35 s^–1^ for the VR (Corridors: 1.64 ± 0.29 s^–1^, Ascending stairs: 2.28 ± 0.49 s^–1^, Descending stairs: 2.21 ± 0.70 s^–1^) and 3.46 ± 0.18 s^–1^ for the RW (Corridors: 3.48 ± 0.29 s^–1^, Ascending stairs: 3.63 ± 1.45 s^–1^, Descending stairs: 3.27 ± 1.55 s^–1^). When computing an ANOVA on the per-participant saccade rates with factors world and sector, we found a significant effect for the factor world [*F*(1,7) = 25.93, *p* = 0.001], but not the factor sector [*F*(2,14) = 0.91, *p* = 0.426] with no significant interaction [*F*(2,14) = 0.63, *p* = 0.545, ε_GG_ = 0.58].

### Dwell Time on Navigational Aids

When walking through corridors, navigation chairs were visible almost continuously, be it at the far end or nearby. However, when ascending or descending stairs, participants did not need to be directed in the proper direction due to lack of directional options. Chairs were therefore rarely within view when passing through those sectors, forcing us to limit this analysis to the corridor sectors. Average gaze dwell time on navigational chairs was 1.8% (SD = 1.4%) of the overall time spent on the walking route for the RW and 3.5% (SD = 2.3%) for VR. This difference was not significant [paired *t*-test, *t*(7) = −1.61, *p* = 0.151]. Average overall gaze-to-chair distance in the virtual world was 13.2° (SD = 5.8°) and in the RW 11.8° (SD = 3.3°). The difference was not significant [paired *t*-test, *t*(7) = 0.59, *p* = 0.570].

## Discussion

In the present study, we investigated how well gaze behavior in the RW can be approximated by measuring gaze in a high-fidelity VR setting. For basic measures like eye position and its spread, we found that differences between sectors (corridors, ascending stairs, and descending stairs) translated from the RW to the virtual setting, with little difference between the worlds.

### Comparison Between VR and the RW

The appearance of the simulated environment of the VR in principle cannot match the RW in all completeness. Focusing on the visual aspects of the VR employed in this study, the visual resolution of the VR system may be very high compared to previously available systems; however, it is still significantly lower than the resolution of the human visual system. The virtual copy of the chosen building, while implemented in great detail (see Supplementary Material), still cannot capture the richness of visual features found in the RW. As a result, a person immersed in the VR will generally be able to tell that they are not looking at the RW. The greatest benefit of the VR is the high degree of control offered by the artificial nature of the virtual surroundings. Environmental factors like the weather or third parties passing through the scenario will not affect the VR, unless desired so by the experimenter. An artificial environment has no practical size limit, and allows for arbitrary (near) real-time manipulations that would be impossible or dangerous in the RW.

### Navigation in VR

In the RW, participants were required to actively walk through the setting. Natural walking behavior can support immersion in the VR (Aldaba and Moussavi, 2020; Cherep et al., 2020; Lim et al., 2020). Navigational self-localization in VR is generally enhanced if participants are allowed to move naturally while immersed in the VR (Klatzky et al., 1998; Aldaba and Moussavi, 2020). The most obvious restriction here was the need for the participants to stay physically within the range of the VR tracking range, while still promoting natural navigational behavior. However, the integration of complex VR settings with treadmills remains challenging, as it requires real-time feedback from motion capture to avoid latencies that disturb immersion. Moreover, walking in such settings is usually restricted to a small range or one linear dimension, as omnidirectional treadmills are far from widespread use as compared to the off-the-shelf head mounted display used here. Technical limitations therefore required us to keep participants within the tracking range of the VR equipment. Hence, we designed the navigation in VR as natural as possible, while participants physically remained within the tracking range, without requiring a VR cave the same size as the real-world building or a multi-directional treadmill. We exploited the observation that being able to orient the body physically appears to be important for immersion in the VR even in the absence of actual walking movement (Cherep et al., 2020; Lim et al., 2020). The solution developed here utilized the system controller, held close to the body, to orient the virtual body of the participant by orienting their real-world bodies in their chosen walking direction. Forward motion was controlled by pressing a button on the controller. This presents a solution to the navigation problem that minimizes the difference to the RW (Klatzky et al., 1998; Waller et al., 2004; Cherep et al., 2020; Lim et al., 2020), as orientation and navigation are still very intuitive and natural, aside from the lack of actual translational (bipedal) movement. Indeed, the analysis of the time spent looking at navigational aids (the chairs with arrows placed along the route) failed to find any significant difference between VR and RW, suggesting that there was likely no principled difference in navigational demands. The analysis of head movement data in VR shows participants moved their head down when negotiating stairs. Stairs are situations where enhanced control of foot placement would be required in the RW, but is not physically necessary in VR. The presence of said head movements is one more point suggesting that the presentation of the virtual world was convincing enough to encourage behavior that would be plausible also in the RW. The naturalness of the navigational solution may also have helped to avoid simulator sickness (no incidences were reported by our participants), which can otherwise be a problem when moving in virtual realities (Golding and Gresty, 2005; Dużmańska et al., 2018).

A simple extension to this approach would be to physically attach the controller to the body of the participant, possible at the hip, which would free one hand and allow for an even more natural posture during exposure to the VR. Our participants did not report any subjective difficulties with the employed method of navigation, and there was no occurrence of cyber sickness. The usage of the standard VR controller for this purpose helps not only to reduce the cost of acquisition of the VR setup, but also cuts down the complexity of the required software development, which will facilitate further experimentation in the future. Generally, while current methods of navigating a virtual environment differ in many cases from the natural means of bipedal movement, this may also offer new chances and opportunities, e.g., in medical rehabilitation training, where patients may be unable to execute the full range of movements available to healthy controls.

### Gaze in VR and RW

Drift – i.e., a growing offset between actual and measured gaze direction that applies uniformly to the whole measured field – is a significant and well-documented factor in head-mounted eye tracking equipment (Sugano and Bulling, 2015; Müller et al., 2019); indeed, the absolute drift in our experiments was quite substantial as compared to stationary eye-tracking equipment. However, there was no significant bias in drift direction for either VR or RW, as well as no significant difference between the RW and VR. This suggests that the results presented here were not systematically affected by changes in position of the measurement equipment during the course of the experiment. Moreover, all measures but the eye-in-head direction, are by construction insensitive against these drifts. Where we *did* consider eye-in-head directions, especially in the gaze-distribution maps of figure 7, the sizes of the observed patterns were large compared to the effects of drift, such that drift is unlikely to have affected these patterns qualitatively. This also applies to individual differences among these gaze maps, which are substantial, a pattern consistent with previous observations on natural scene viewing (e.g., Yarbus, 1967; de Haas et al., 2019).

When real-world gaze allocation is compared to standard laboratory eye-tracking settings, profound differences are found, in particular with respect to gaze in direction of the ground (’t Hart et al., 2009). However, there are multiple differences between walking through the RW and watching the same visual input on a screen: the visual input on the screen is limited in visual field and resolution, head and body are restrained and there is no need to actively navigate or walk through the environment. To isolate the component of safe walking from the other differences, we here attempted to approximate the natural situation with respect to its visual appearance and its navigational requirements as closely as technically possible in VR. As expected from real-world studies (’t Hart and Einhäuser, 2012), we found profound differences between different terrains (sectors) for nearly all of the measures tested. One might have also expected differences between the worlds or an interaction of the world with terrain (if the VR had been perceived as entirely unconvincing by the participants, gaze patterns in VR might have differed less between the different sectors than in RW). In particular, one might assume that the additional requirement to place one’s feet carefully in the RW as compared to VR (Matthis et al., 2018; Kopiske et al., 2020; Thomas et al., 2020) would be accompanied by significant changes in gaze behavior, especially when negotiating the stairs. Surprisingly, however, we found no interactions between the factors world and sector for any of the measures tested. Effects of the world were found for the vertical gaze direction in eye-in-head coordinates, the vertical head orientation in VR, the number of saccades made and a subtle difference in the angular distribution of gaze-direction changes. The direction of the former effect – gaze was lower in VR on average than in the RW – was contrary to expectations (’t Hart et al., 2009; ’t Hart and Einhäuser, 2012): one would expect virtual locomotion to require fewer looks to the ground where the information for foot placement is gathered in the RW (Marigold and Patla, 2007) and also during actual walking in VR (Kopiske et al., 2020). However, this effect is likely explained by headset placement and absent (numerically even reversed) when gaze is referenced to the horizon. As a measure that is insensitive to offsets in the headset placement, we quantified the spread of eye movements by using the IQR (Figure 6). As before, we found significant differences only between the sectors, not between the worlds, and importantly no interaction between the factors. This underlines the observation that the differences between sectors translate well from the RW to the VR, and – for our setting – differences between the worlds are minute. The differences in angular distribution of gaze-direction changes between the worlds are also subtle, provided the comparably large differences found between different real-world environments (Einhäuser et al., 2007), which in turn are comparable to the differences between sectors in the present study. It is tempting to speculate that similar factors generate the differences and are related to the requirements of the environment, with more liberal (less navigation-driven) exploration in the VR generating more cardinal eye movements. Generally, fixations and smooth pursuit are not trivial to tell apart in head-free scenarios, as what looks like pursuit in gaze angle velocities may indeed be a fixation on a physically stationary object in the presence of head movements. Additionally, a physically stationary object such as a light switch on a wall may move through the observer’s field of view on a path consistent with the optical flow as the observer moves forward. The gaze velocity analysis indicates relatively more saccades in the RW, suggesting more exploratory saccades, perhaps due to a more navigation-driven exploratory behavior. This is also supported by the relative increase of higher velocities in the gaze velocity analysis, which in turn finds more slow eye movements in the VR. This would be well explained by an increased number of optic flow linked fixations as a counterpoint to the increased number of saccades in RW.

For the “T-shape” previously described in the RW (Calow and Lappe, 2008; ’t Hart et al., 2009), we find a trend to an interaction between world and sector, so we cannot exclude that differences between VR and RW will start to emerge when more sophisticated measures or more difficult terrain (as compared to the smooth floor surface of an office building) are concerned. Explicitly modeling difficult and irregular terrain in VR will therefore become an interesting line for further research (cf. Kopiske et al., 2020).

## Conclusion

In summary, we found surprisingly little difference between gaze behavior in VR and RW for our setting; to the contrary, virtual locomotion seems to capture the major differences between different environmental constraints (the factor “sector” in our experiment) remarkably well. The effects of world (VR vs. RW) we found were either well-explainable by equipment particularities, as for the vertical eye-in-head position, or subtle compared to previously reported differences between different real-world settings, as in the case of the cardinal preferences. This opens up an avenue of possibility for research that would previously have been possible only in real-world settings, but with the enhanced control over environmental factors offered by VR that would otherwise be largely left at random. Gaze analysis in life-like settings, but still under highly controlled conditions, has therefore now become a tangible reality. Remaining factors that may affect the depth of immersion and thus also the similarity of the gaze behavior in simulated environments may be addressed through improved VR devices, such as treadmills to allow for even more realistic navigation (Kopiske et al., 2020) or improvements in available computational power for even more visual details.

## Data Availability Statement

The raw data supporting the conclusions of this article will be made available by the authors, without undue reservation.

## Ethics Statement

All procedures were evaluated by the applicable ethics board (Ethikkommission der Fakultät HSW, TU Chemnitz) who ruled that no in-depth evaluation was necessary (case-no.: V-274-PHKP-WET-Augenb-11062018). The participants provided their written informed consent to participate in this study.

## Author Contributions

All authors conceived the study, designed the experiment, and reviewed the manuscript. SF created the virtual reality model. SF and WE collected the data. JD and SF analyzed the data. JD and WE wrote the manuscript.

## Conflict of Interest

The authors declare that the research was conducted in the absence of any commercial or financial relationships that could be construed as a potential conflict of interest.

## Footnotes

1 High quality versions of the videos in the Supplementary Material can be found at https://doi.org/10.6084/m9.figshare.14553738 and https://doi.org/10.6084/m9.figshare.14553759.
